# Ag120-Mediated Inhibition of ASCT2-Dependent Glutamine Transport has an Anti-Tumor Effect on Colorectal Cancer Cells

**DOI:** 10.3389/fphar.2022.871392

**Published:** 2022-03-28

**Authors:** Wei Yu, Jianwen Huang, Qichao Dong, Wenting Li, Lei Jiang, Qian Zhang, Li Sun, Shengtao Yuan, Xu He

**Affiliations:** ^1^ Zhuhai Interventional Medical Center, Zhuhai Precision Medical Center, Zhuhai People’s Hospital, Zhuhai Hospital Affiliated with Jinan University, Jinan University, Zhuhai, China; ^2^ Jiangsu Key Laboratory of Drug Screening, China Pharmaceutical University, Nanjing, China; ^3^ Department of General Surgery, Zhuhai People’s Hospital (Zhuhai Hospital Affiliated with Jinan University), Zhuhai, China

**Keywords:** ASCT2, AG120, CRC, glutamine metabolism, tumor proliferation

## Abstract

Metabolic reprogramming is considered to be a hallmark of cancer, and increased glutamine metabolism plays an important role in the progression of many tumors, including colorectal cancer (CRC). Targeting of glutamine uptake *via* the transporter protein ASCT2/*SLC1A5* (solute carrier family 1 member 5) is considered to be an effective strategy for the treatment of malignant tumors. Here, we demonstrate that Ag120 (ivosidenib), a mutant isocitrate dehydrogenase 1 (IDH1) inhibitor approved for the treatment of certain cancers, acts as an ASCT2 inhibitor in CRC cells. Ag120 blocked glutamine uptake and metabolism, leading to reduced cell proliferation, elevated autophagy, and increased oxidative stress in CRC cells *in vitro* and *in vivo*, potentially *via* the ERK and mTOR signaling pathways. These effects occurred independently of mutant IDH1 activity and were supported by experiments with ASCT2-depleted or -overexpressing cells. These data identify a novel mechanism of Ag120 anti-tumor activity and support further exploration of ASCT2 inhibitors for cancer therapy.

## Introduction

Deregulation of cell metabolism, also known as metabolic rewiring, is a hallmark of cancer and is a response to the increased demands for energy and materials needed to support the growth phenotypes of cancer cells (Hanahan and Weinberg, 2000; San-Millán and Brooks, 2017). L-glutamine is a crucial amino acid for protein synthesis and energy generation by many tumors, including colorectal cancer (CRC) (Deberardinis and Cheng, 2010; Noel et al., 2010). Typically, transportation of glutamine into cells is mediated by amino acid carrier proteins, particularly solute carrier family 1 member 5 (*SLC1A5*), also known as alanine, serine, cysteine-preferring transporter 2 (ASCT2) (Wang et al., 2015). *SLC1A5* is highly expressed in many cancers, including CRC (Deberardinis and Cheng, 2010), and plays an important role in supplying glutamine for energy production, autophagy, redox homeostasis, and activation of mTOR signaling, thereby promoting tumor growth (Hassanein et al., 2013; Willems et al., 2013; Huang et al., 2014; Marzi et al., 2016; van Geldermalsen et al., 2016). Inhibition of ASCT2 protein and glutamine starvation represents a promising strategy for cancer therapy. Consistent with this, many studies have investigated the effects on cancer growth of ASCT2 inhibition with small molecule compounds such as V9302 (Schulte et al., 2018), l-γ-glutamyl-p-nitroanilide (GPNA) (Esslinger et al., 2005), and benzylserine (Grewer and Grabsch, 2004), and with ASCT2-specific monoclonal antibodies (Hara et al., 2020). However, each of these approaches has some limitations such as high toxicity and poor solubility (Grewer and Grabsch, 2004; Esslinger et al., 2005; Schulte et al., 2018; Hara et al., 2020). And there is an urgent need to continue the search for new ASCT2 inhibitors for the treatment of cancer.

Ag120, also known as ivosidenib, is a small molecule inhibitor of mutant isocitrate dehydrogenase (IDH1mt). The driver mutations in IDH1 abolish its normal function in the conversion of isocitrate to α-ketoglutarate (α-KG) and instead confer gain-of-function activity that results in the production of the oncometabolite 2-hydroxyglutarate (2-HG) (Lucia et al., 2017; Marina et al., 2021). In the United States, ivosidenib was approved for the treatment of IDH1mt acute myeloid leukemia in 2018 and for IDH1mt cholangiocarcinoma in 2021, and it is currently in clinical trials for other cancers such as advanced hematological malignancies (NCT02074839) and advanced solid tumors (NCT02073994) harboring IDH1mt (Lucia et al., 2017; Marina et al., 2021). Ag120 mainly targets tumor cells by inhibiting IDH1mt-mediated production of 2-HG, thereby blocking of tumor progression (Pollyea et al., 2014; Fan et al., 2015). Interestingly, Ag120 inhibits the proliferation of a subcutaneous grade 3 IDH1mt glioma by 52% through an unknown mechanism (Brandon et al., 2017). Therefore, it seems likely that the anti-tumor effects and mechanisms of action of Ag120 are incompletely understood, and continued study of this compound is warranted. To date, the anti-CRC effects of Ag120 have not been investigated and its mechanisms of action other than *via* IDH1 mt are also unknown.

In the present study, we report for the first time that Ag120 is an ASCT2 inhibitor in CRC cells and suppresses tumor growth *via* inhibition of glutamine uptake and metabolism. This work not only has important implications for broadening the potential indications for Ag120 but also identifies ASCT2 as a potential anti-cancer therapeutic target of Ag120.

## Materials and Methods

### Cell Culture and Reagents

The human CRC cell lines HCT116 and HT29 were obtained from NEWGAINBIO (Wuxi, China) and were authenticated using short tandem repeat analysis (Genetic Testing Bio-technology Corporation, Suzhou) to exclude possible contamination. CRC cells were maintained in McCoy’s 5A medium containing 10% fetal bovine serum at 37°C in a 5% CO_2_ humidified atmosphere. Ag120 and the ASCT2 inhibitor V9302 were obtained from Selleck and were resuspended in phosphate-buffered saline (PBS) for experiments.

### Computational Screening

The datafile for the structure of the human ASCT2 protein [Protein Data Bank (PDB): 5LLM]was downloaded from PDB (http://www.rcsb.org/). All heterogeneous atoms were removed for subsequent molecular docking. The PDB file (5LLM) was converted to the PDBQT format for macromolecules before virtual screening. The grid (ligand docking search space) was located and maximized. Autodock Vina 1.1.2 was used for molecular docking. Protein–ligand interactions were visualized using PyMOL version 1.7.4.5.

### Surface Plasmon Resonance

Binding of Ag120 to purified human ASCT2 protein (ACROBiosystems, Beijing, China) was measured by SPR using a Biacore T200 system (General Electric, Sweden). Binding was tested in the presence of two-fold serial dilutions of Ag120 from 0.15625 to 10 µM. Each sample was analyzed in triplicate.

### Construction of CRC Cell Lines With Stable ASCT2 Knockdown or Overexpression

HCT116 and HT29 cells were seeded in tissue culture dishes and grown for approximately 24 to reach 50–60% confluence. The medium was then changed to McCoy’s 5A containing 30 μg/ml polybrene and the appropriate lentiviruses encoding control or ASCT2 shRNAs (sh *SLC1A5*#1: 5′-GCT​TGG​TAG​TGT​TTG​CCA​TCG-3′; sh *SLC1A5*#2: 5′-GGA​TGT​GGG​TTT​ACT​CTT​TGC-3′; sh NC: 5′-TTC​TCC​GAA​CGT​GTC​ACG​T-3′; Public Protein/Plasmid Library, Nanjing, China) or overexpression vectors (pLenti-CMV-GFP-Puro-*SLC1A5*; Public Protein/Plasmid Library, Nanjing, China) were added and incubated at 37°C for 24 h. The medium was exchanged for fresh McCoy’s 5A medium and the cells were further cultured for an additional 24 h. Stable ASCT2 knockdown (ASCT2-KD) or ASCT2-overexpressing (ASCT2-OE) cell lines were selected by growth in medium containing 10 µM puromycin. Efficient knockdown or overexpression was verified by RT-qPCR and immunoblotting.

### Colony-forming Assay

CRC cells were seeded in 6-well culture plates at 10^3^ cells/well and treated with Ag120 or V9302 for 10 days (wild-type cells) or 14 days (ASCT2-KD or ASCT2-OE cells). At the end of the incubation, the cells were stained with crystal violet solution (Beyotime, Jiangsu, China) and the number of colonies was counted manually.

### Colorimetric Cell Proliferation Assay

Cells were seeded in 96-well plates at 2 × 10^4^ cells/well and incubated for 72 h. Every 24 h, cell numbers were determined using a Cell Counting Kit-8 (CCK-8; Beyotime, Jiangsu, China) according to the manufacturer’s instructions.

### EdU Incorporation

To assess proliferation by incorporation of the fluorescent nucleoside analog 5-ethynyl-2′-deoxyuridine (EdU), CRC cells were seeded in 96-well plates at 3 × 10^4^ cells/well and incubated for 72 h. EdU incorporation was assessed using an EdU Staining Proliferation kit (RuiBo, Suzhou, China) according to the manufacturer’s instructions.

### Immunoblotting

CRC cells were incubated with Ag120 or V9302 for 48 h and processed for immunoblotting as previously described (Schulte et al., 2018). Primary antibodies against β-actin, ASCT2, mTOR, phosphorylated (p)-mTOR, P70S6K, p-P70S6K, ERK1, p-ERK1, IDH1wt, LC3, LAMP1, ATG7, ATG5, and beclin-1 and a secondary FITC-conjugated goat anti-rabbit IgG (H + L) antibody were purchased from ABclonal Biotechnology (Wuhan, China). Horseradish peroxidase (HRP)-conjugated anti-mouse IgG was purchased from ABclonal Biotechnology (Wuhan, China) and anti-rabbit IgG secondary antibodies were purchased from Cell Signaling Technology (Beverly, MA, United States). Protein bands were visualized using enhanced chemiluminescence reagents (Millipore).

### Quantitative Reverse-Transcription PCR (RT-qPCR)

Total cellular RNA was isolated from cells or tissues using TRIzol reagent (Vazyme, Jiangsu, China) and RNA was reverse transcribed using a Revert Aid First Strand cDNA Synthesis Kit (Vazyme). qPCR was performed using SYBR GREEN master mix (Vazyme) on a Bio-Rad CFX-96 fluorescence quantitative PCR instrument. mRNA levels of the genes of interest were standardized with an internal control (18s rRNA). Primer sequences are provided in Table 1.

**TABLE 1:** Primer sequences for quantitative RT-PCR.

Gene	Sequences
SLC1A5	Forward Sequence: TCC​TCT​TCA​CCC​GCA​AAA​ACC​C
—	Reverse Sequence: CCA​CGC​CAT​TAT​TCT​CCT​CCA​C

### Mouse Xenograft Experiments

Groups (n = 5) of female athymic BALB/c nude mice (5–6 weeks of age, body weight 18–22 g) were obtained from Charles River (ZheJiang, China). Subconfluent HCT116 cells were collected, resuspended in serum-free medium at 10^6^ cells/100 μl, and injected subcutaneously into one flank. After 7 days, the animals were injected intraperitoneally with the indicated doses of Ag120, V9302, or vehicle (PBS) in 100 µl/injection. Animal care and experimental protocols were approved by the Animal Care Committee of JINAN University. Animals were treated appropriately and used in a scientifically valid and ethical manner.

### BrEdU Incorporation Assay

Four hours after the final injection of vehicle, Ag120 or V9302, the mice were injected intraperitoneally with 100 µl of 1 mg/ml bromodeoxyuridine (BrEdU) labeling reagent (RuiBO) and sacrificed 4 h later. The tumors were excised, frozen, and cryosectioned. Sections were incubated with 10 µg of mouse anti-BrEdU primary antibody (ABclonal Biotechnology, Wuhan, China) for 12 h at 4°C and then with a rhodamine red-labeled goat anti-mouse IgG secondary antibody (Invitrogen Molecular Probes, Carlsbad, CA) for 2 h at 20°C. Sections were counterstained with the DNA-binding dye 4′,6-diamidino-2-phenylindole (DAPI). Cells were imaged on an inverted fluorescence microscope and photographs were obtained and scanned into ImageJ software. BrEdU-incorporating and proliferating cells were quantified using high-magnification photographs.

### Glutamine Uptake

Cells were cultured overnight in 3.5-ml culture dishes at 2 × 10^6^/dish. Cells were transferred to PBS containing 1 mg/mL d-glucose and 0.11 mg/ml sodium pyruvate and incubated with Ag120 or V9302 for 2 h L-glutamine (2 mM) was then added to the cells for an additional 10 min and the cells were collected, washed, and lysed. Intracellular glutamine concentrations were measured using a glutamine assay kit (Abnova, United States) according to the manufacturer’s instructions. Luminescence was measured using a FilterMax F3 microplate reader. The data were normalized to total protein levels.

### Glucose Uptake

Cells were seeded in a 6-well plate at 5 × 10^5^ cells/well. The cells were glucose-starved by preincubation in 1 ml Krebs-Ringer-Phosphate-HEPES buffer containing 2% bovine serum albumin and Ag120 or V9302 for 40 min, and 10 µl of 10 mM 2-deoxyglucose (Sigma Aldrich, St. Louis, MO, United States) was then added for an additional 20 min. At the end of the incubation, the cells were collected, washed, and lysed, and glucose concentrations in the supernatants were measured with a Glucose Uptake kit (Abcam, Cambridge, United Kingdom) according to the manufacturer’s instructions.

### Intracellular Metabolite Assays

CRC cells were treated for 48 h with Ag120 and then processed for measurement of intracellular levels and analyzed for intracellular levels of NAD^+^/NADH, GSH, and ATP using kits from Beyotime; for glutamate, glucose, and α-KG using kits from Abcam (Cambridge, United Kingdom); and for d-2-hydroxyglutarate (2-HG) levels using a colorimetric assay kit from BioVision (Bioptics, Tucson, AZ, USA).

### Immunohistochemistry

Tumors were excised from the mice within 4 h of the final vehicle, Ag120, or V9302 administration, and then fixed in 10% formalin for 24 h and stored in 70% ethanol in PBS at 4 °C. Tissues were sectioned (5 µm thickness) and stained with primary antibodies against ASCT2 (Cell Signaling Technology, #8057S), Ki67 (Abclonal #A2094), or LC3 (Cell Signaling Technology, #12741S) followed by anti-rabbit IgG secondary antibodies (Cell Signaling Technology, Beverly, MA, United States). Tissues were imaged using an inverted fluorescence microscope at ×20 magnification.

### Transmission Electron Microscopy

TEM was used to visualize autophagic vesicles and mitochondrial morphology. CRC cells were treated with Ag120 and V9302 for 72 h and fixed in glutaraldehyde (Sigma). Ultrathin sections were prepared using a Sorvall MT5000 microtome and stained with lead citrate and/or 1% uranyl acetate. Sections were visualized using a Philips EM420 electron microscopy.

### Detection of Lysosomes and Autophagosomes

Lysosomes and autophagosomes were visualized using the fluorescent probes LysoTracker and monodansylcadaverine (MDC), respectively. Cells were seeded in 6-well plates at 5 × 10^5^/well and grown overnight. Ag120 or V9302 was added and the cells were incubated for 72 h. The culture medium was then removed and the cells were processed using a LysoTracker staining kit (Beyotime), or an MDC staining kit (Solarbio, Beijing, China) according to the manufacturers’ instructions. Cells were visualized using an inverted fluorescence microscope.

### Statistical Analysis

Data are presented as the mean ± standard deviation (SD). Multiple group means were compared using two-way analysis of variance followed by the Student–Newman–Keuls multiple comparison test, and two group means were compared using Student’s t-test. A *p* value < 0.05 was considered to be statistically significant.

## Results

### Ag120 Binds to Purified Human ASCT2 Protein and Modulates Energy Metabolism in CRC Cells

**FIGURE 1:**
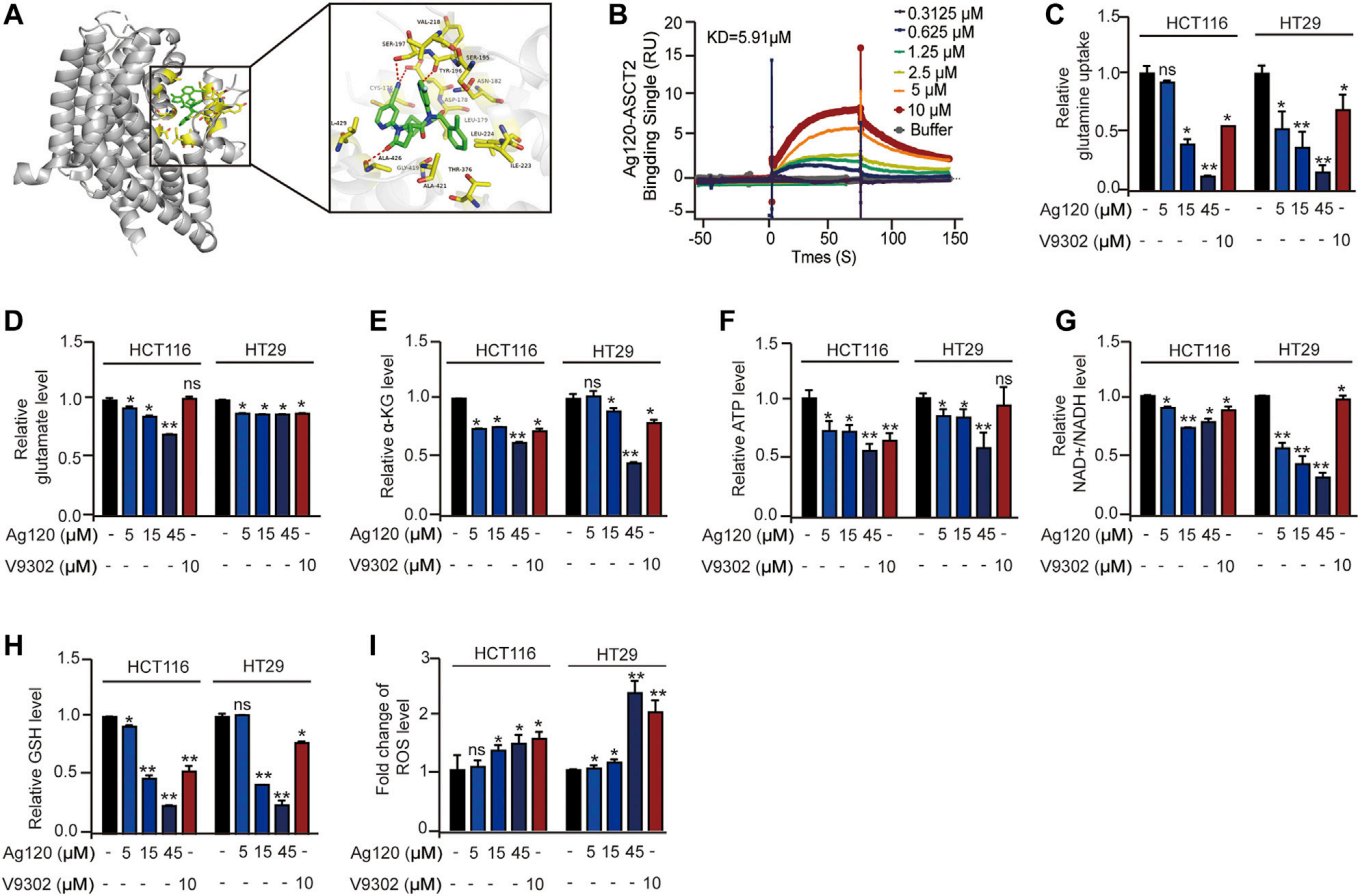
Ag120 binds to isolated ASCT2 protein and inhibits the metabolism of CRC cells. **(A)** Modeling of Ag120 (green stick) docking superimposed on ASCT2 protein (PDB: 5LLM), with the interacting ASCT2 amino acids represented by orange sticks. Inset shows higher magnification. **(B)** SPR analysis of the binding affinity of Ag120 for purified ASCT2. **(C–I)** Intracellular glutamine **(C)**, glutamate **(D)**, α-KG **(E)**, ATP **(F)**, NAD^+^/NADH **(G)**, GSH **(H)**, and ROS **(I)** levels in HCT116 and HT29 cells treated with Ag120 or V9302 for 72 h. Mean ± SD of at least three independent experiments. *p < 0.05, **p < 0.01 by Student’s *t*-test.

By employing the crystal structure of ASCT2 to perform molecular docking modeling, we find the potential interaction between Ag120 and ASCT2 protein (Canul-tec et al., 2017). As shown in Figure 1A, Ag120 binds to ASCT2 through amino acids CYS175, LEU179, ASN182, SER195, SER197, VAL218, ILE223, LEU224, THR376, GLY419, ALA421, ALA426, and VAL429 (Figure 1A). Consistent with this, SPR experiments demonstrated a binding affinity of 5.91 µM between purified human ASCT2 protein and Ag120 (Figure 1B) and V9302 is regarded as the positive control (Supplementary Figure S1A) (Schulte et al., 2018). To examine the pharmacological effects of Ag120, we incubated two human CRC cell lines, HCT116 and HT29, with various concentrations of Ag120 or with 10 µM of V9302, a previously described ASCT2 inhibitor (Schulte et al., 2018) for 72 h and then analyzed the intracellular concentrations of glutamine, α-KG, ATP, NAD^+^/NADH, glutathione (GSH), and reactive oxygen species (ROS). Ag120 was found to significantly decrease intracellular levels of glutamate, α-KG, ATP, and NAD^+^/NADH in both cell types (Figures 1D–G), whereas glucose concentrations were significantly elevated (Supplementary Figure S1B), possibly as a compensation mechanism. Moreover, the block of glutamine uptake could cause the reduce of GSH and increase of ROS thereby inducing oxidative stress (Schulte et al., 2018; Hara et al., 2020). Ag120 altered the redox balance in CRC cells, as reflected by the significant depletion of GSH levels and elevation of ROS levels compared with untreated cells (Figures 1H,I). These changes were also observed with the known ASCT2 inhibitor V9302, although V9302 appeared to be the more effective inhibitor of the two (Figures 1D–I). Collectively, these results demonstrated that Ag120 binds to ASCT2, inhibits glutamine metabolism and energy production, and induces oxidative stress in CRC cells.

### Ag120 Inhibits Proliferation in CRC Cells

**FIGURE 2:**
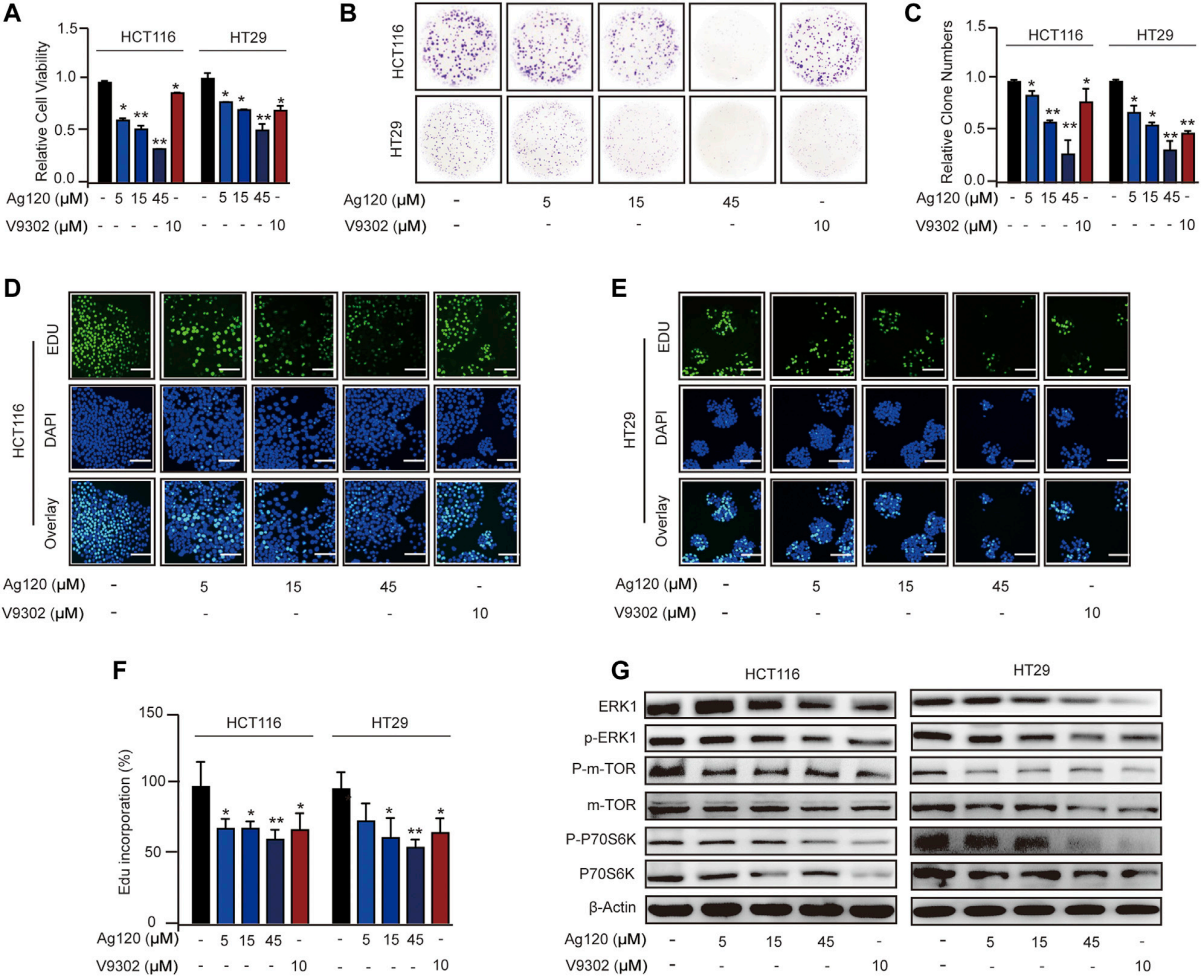
Ag120 inhibits CRC cell proliferation *via* suppression of ERK and mTOR signaling pathways. **(A)** Quantification of HCT116 and HT29 cells after culture for 72 h with Ag120 or V9302 was performed using a CCK-8 kit. **(B,C)** Plate images **(B)** and quantification **(C)** of colony formation by HCT116 and HT29 cells cultured for 10 days with Ag120 or V9302. **(D–F)** Fluorescence microscopy images **(D,E)** and quantification **(F)** of EdU incorporation in HCT116 and HT29 cells cultured for 3 days with Ag120 or V9302. Scale bars, 50 µm. **(G)** Immunoblot analysis of ERK1, p-ERK1, mTOR, p-mTOR (S2448), P70S6K, and p-P70S6K expression in CRC cells treated with Ag120 or V9302 for 72 h. Mean ± SD of at least three independent experiments. ns, no significance; *p < 0.05, **p < 0.01 by Student’s *t*-test.

To determine whether Ag120 exhibited an anti-tumor effect on CRC cells, we analyzed cell proliferation, colony formation, and DNA synthesis. Incubation of CRC cells with Ag120 significantly and dose-dependently decreased the number of viable cells present after 72 h (Figure 2A) and the number of colonies formed after 10 days (Figures 2B,C). These effects were confirmed by demonstrating a significant reduction in incorporation of the fluorescent nucleoside EdU into CRC cell DNA after 72 h treatment with Ag120 (Figures 2D–F). To identify the potential mechanism of action of Ag120, we performed immunoblot analysis of key components of the ERK and mTOR signaling pathways in HCT116 and HT29 cells. Ag120 treatment for 72 h resulted in decreased expression of ERK1, phosphorylated (p)-ERK1, mTOR, p-mTOR (S2448), P70S6K, and p-P70S6K (Figure 2G; Supplementary Figure S2) which is related to tumor proliferation (van Geldermalsen et al., 2016; Schulte et al., 2018). Collectively, these results suggest that Ag120 exhibits a significant anti-tumor effect in CRC that may be mediated by inhibition of the mTOR and ERK1 signaling pathways.

### Ag120 Induces Autophagy in CRC Cells

**FIGURE 3:**
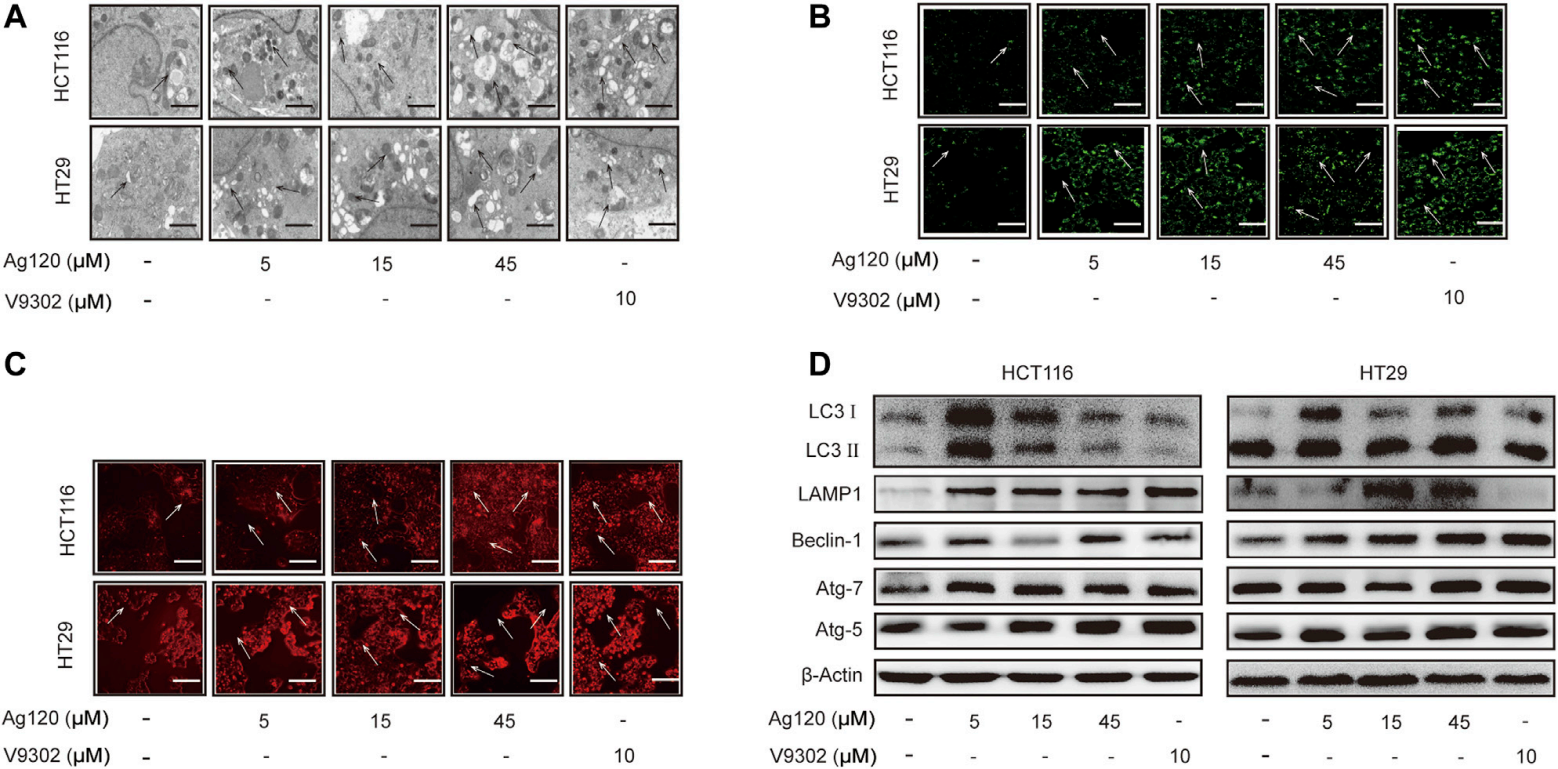
Ag120 induces autophagy in CRC cells. **(A)** Transmission electron microscopy images of HCT116 cells treated with AG120 or V9302 for 72 h. Scale bars, 1 µm. **(B,C)** Fluorescence microscopy images of CRC cells incubated with Ag120 or V9302 for 72 h and labeled with MDC to visualize autophagic vesicles **(B)** or with LysoTracker to visualize lysosomes **(C)**. Scale bars, 50 µm. **(D)** Immunoblot analysis of LC3, LAMP1, beclin-1, ATG7, and ATG5 expression in CRC cells treated with Ag120 or V9302 for 72 h.

Autophagy has been shown to be elevated upon ASCT2 depletion and glutamine starvation *in vitro* and *in vivo* (Schulte et al., 2018). Therefore, we next investigated whether Ag120 might affect autophagy in CRC. To this end, we performed TEM as well as fluorescence microscopy of cells stained with the fluorescent probes MDC or LysoTracker Red, which preferentially accumulate in autophagosomes and lysosomes, respectively. Ag120 treatment of HCT116 cells for 72 h resulted in the presence of a large number of vacuoles of varying sizes and clear punctate structures in the cytoplasm or perinucleus (Figure 3A), suggesting that Ag120 treatment induced the formation of autophagic vesicles. Ag120 treatment also increased the number of MDC- and LysoTracker Red-stained foci in the cytoplasm of Ag120-treated cells, indicative of autophagosome and lysosome accumulation (Figures 3B,C; Supplementary Figures S2A,B). To probe this further, we examined expression of a number of autophagy-related proteins by immunoblot analysis and found that Ag120 treatment increased the expression of the autophagy markers LC3, beclin-1, ATG7, and ATG5, and of the lysosomal marker LAMP-1 (Figure 3D; Supplementary Figures S3C–H). Taken together, these results demonstrate that Ag120 induces autophagy in CRC cells.

### Ag120 Suppresses the Proliferation of CRC Cells by Inhibiting Glutamine Metabolism

**FIGURE 4:**
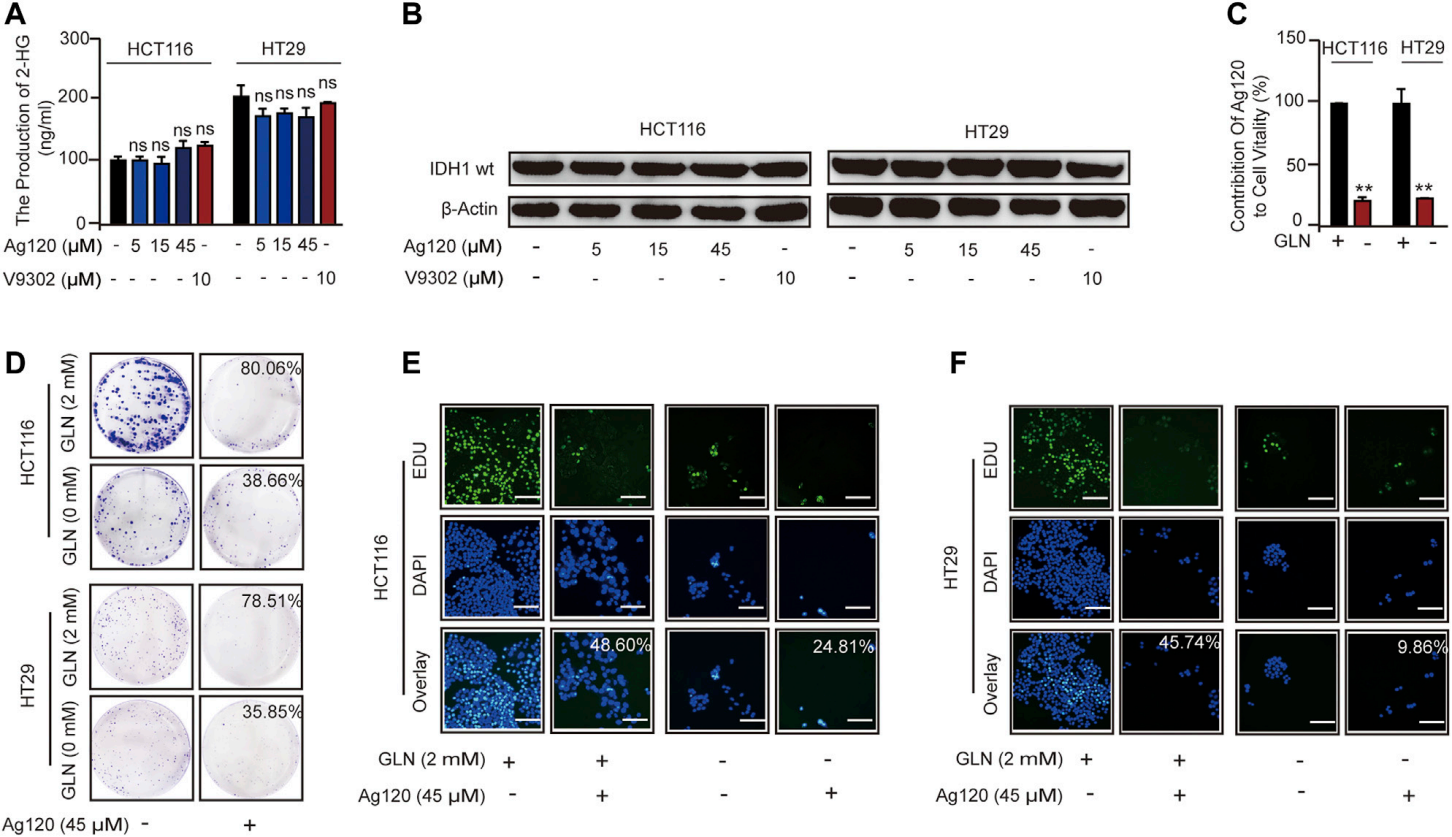
Effects of Ag120 and glutamine depletion on CRC cells. **(A,B)** 2-HG production **(A)** and immunoblot analysis of IDH1mt expression **(B)** in CRC cells treated with Ag120 or V9302 for 72 h. **(C)** Quantification of viable HCT116 and HT29 cells treated with vehicle or Ag120 (45 µM) for 72 h in DMEM medium containing 2 or 0 mM L-glutamine. Cell numbers were measured using a CCK-8 kit. Contribution of Ag120 to cell viability was calculated based on the data in Supplementary Figure S3A. **(D)** Colony formation by CRC cells after 10 days of treatment with 0 or 45 µM Ag120 in 0 or 2 mM L-glutamine-containing medium. **(E,F)** Fluorescence microscopy images of EdU incorporation in HCT116 **(E)** and HT29 **(F)** cells treated for 72 h with 0 or 45 µM ASCT2 in 0 or 2 mM L-glutamine-containing medium. Scale bars, 50 µm. Mean ± SD of three experiments. *p < 0.05, **p < 0.01 by Student’s *t*-test.

We next asked whether the anti-tumor effects of Ag120 in CRC cells were mediated *via* inhibition of glutamine metabolism alone or whether IDH1mt inhibition might also contribute. Ag120 might also have an effect on the IDH1wt in tumor cells (Pollyea et al., 2014; Fan et al., 2015). In addition, IDH-mutated tumor cells could also cause abnormalities in the glutamine metabolic pathway (Lucia et al., 2017; Marina et al., 2021). However, we examined the effects of Ag120 treatment for 72 h on intracellular 2-HG levels and found that the production of 2-HG in CRC cells is about 100–300 ng/ml (Figure 4A), which means CRC cells do not exist IDH1 mutation (Pollyea et al., 2014; Marina et al., 2021). Moreover, we examined the effects of Ag120 treatment for 72 h on intracellular 2-HG levels and on IDH1wt expression and found that concentrations of Ag120 that could inhibit CRC proliferation and promote autophagy had no significant effects on either 2-HG production (Figure 4A) or IDH1wt expression (Figure 4B; Supplementary Figure S4A), suggesting that Ag120 effects were independent of IDH1mt and IDH1wt in these CRC cells. We next examined the effects of Ag120 in cells grown in glutamine-depleted medium. As expected, depletion of extracellular glutamine significantly reduced the growth of CRC cells over 72 h (Figure 4C; Supplementary Figure S4B). However, Ag120 exhibited a more significant anti-tumor effect in 2 mM glutamine-containing compared with glutamine-depleted medium, as shown by colony formation and EdU incorporation assays (Figures 4D–F; Supplementary Figures S4C–F). These results suggested that Ag120 inhibition of CRC cell proliferation most likely occurs independently of IDH1mt and instead is mediated *via* its effects on glutamine metabolism.

### Ag120 Anti-Tumor Effect is Predominantly Mediated by ASCT2 Inhibition in CRC Cells

**FIGURE 5:**
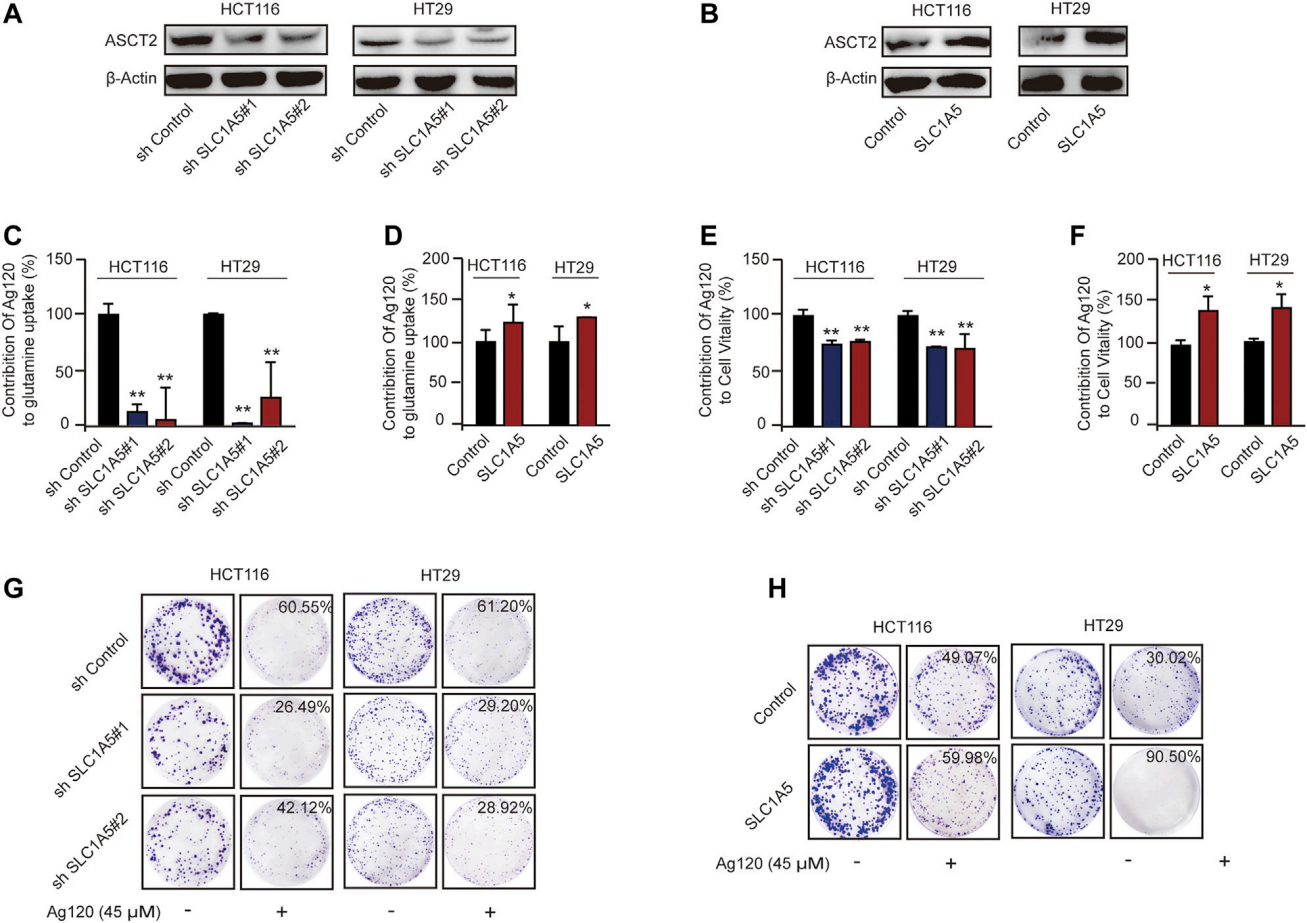
Effects of Ag120 on glutamine metabolism and proliferation in CRC cells following ASCT2/SLC1A5 knockdown or overexpression. **(A,B)** Immunoblot analysis of ASCT2 in HCT116 and HT29 cells stably transfected with ASCT2 shRNA (shSLC1A5#1/shSLC1A5#2) or control shRNA **(A)** or stably transfected with a control or SLC1A5 DNA-lipid overexpression complex **(B)**. **(C,D)** Glutamine uptake in SLC1A5-depleted or SLC1A5-overexpressing HCT116 and HT29 cells treated for 2 h with Ag120 (15 µM). The contribution of Ag120 to glutamine uptake was calculated based on the data in Supplementary Figures S4C,D. **(E,F)** Viability of SLC1A5-depleted or SLC1A5-overexpressing HCT116 and HT29 cells treated for 72 h with Ag120 (45 µM) was quantified using a CCK-8 kit. The contribution of Ag120 to cell viability was calculated based on the data in Supplementary Figures 4E,F. **(G,H)** As described for **(E,F)** except colony formation was measured after 10 days. Mean ± SD of three experiments. *p < 0.05, **p < 0.01 by Student’s *t*-test.

To test this hypothesis further, we constructed HCT116 and HT29 cell lines with stable ASCT2 knockdown (ASCT2-KD) induced by lentivirus-mediated expression of *SLC1A5* shRNA (sh*SLC1A5*#1/sh*SLC1A5*#2) or with stable overexpression of ASCT2 (ASCT2-OE) mediated by transfection of DNA-lipid complexes. We performed immunoblot analysis and RT-qPCR to verify effective ASCT2 silencing or overexpression (Figures 5A,B; Supplementary Figures S5A–D) and then analyzed the tumor cell phenotypes. In keeping with the proposed mechanism of action of Ag120, we found that the inhibitor had different effects in ASCT2-KD and ASCT2-OE CRC cells (Supplementary Figures S5E,F)*.* ASCT2 KD and OE resulted in decreased and increased glutamine uptake, respectively (Supplementary Figures S5E,F). Compared with cells expressing control shRNA, the relative glutamine inhibition rate of ASCT2-KD cells which is calculated based on the data in Supplementary Figure S5E decreased significantly (Figure 5C; Supplementary Figure S5E), and conversely, ASCT2-OE cells exhibited a high rate of glutamine inhibition rate compared with the control cells (Figure 5D; Supplementary Figure S5F).

We next compared the effect of Ag120 on ASCT2-KD and ASCT2-OE cells. CRC cell viability decreased in ASCT-KD cells (Supplementary Figure S5G) and increased in ASCT2-OE cells Supplementary Figure S5H). Additionally, compared with the control cells, the relative inhibition rate of Ag120 which is calculated based on the data in Supplementary Figures S5G,H was decreased in the ASCT-KD cells (Figure 5G; Supplementary Figure S5G) and increased in the ASCT-OE cells (Figure 5F; Supplementary Figure S5H). The effects of Ag120 on colony formation were consistent with these observations (Figures 5G,H; Supplementary Figures S5I–L). These results suggested that Ag120 might exert its anti-tumor effects in CRC cells *via* ASCT2.

### Ag120 Exhibits an Anti-CRC Effect *in Vivo*

**FIGURE 6:**
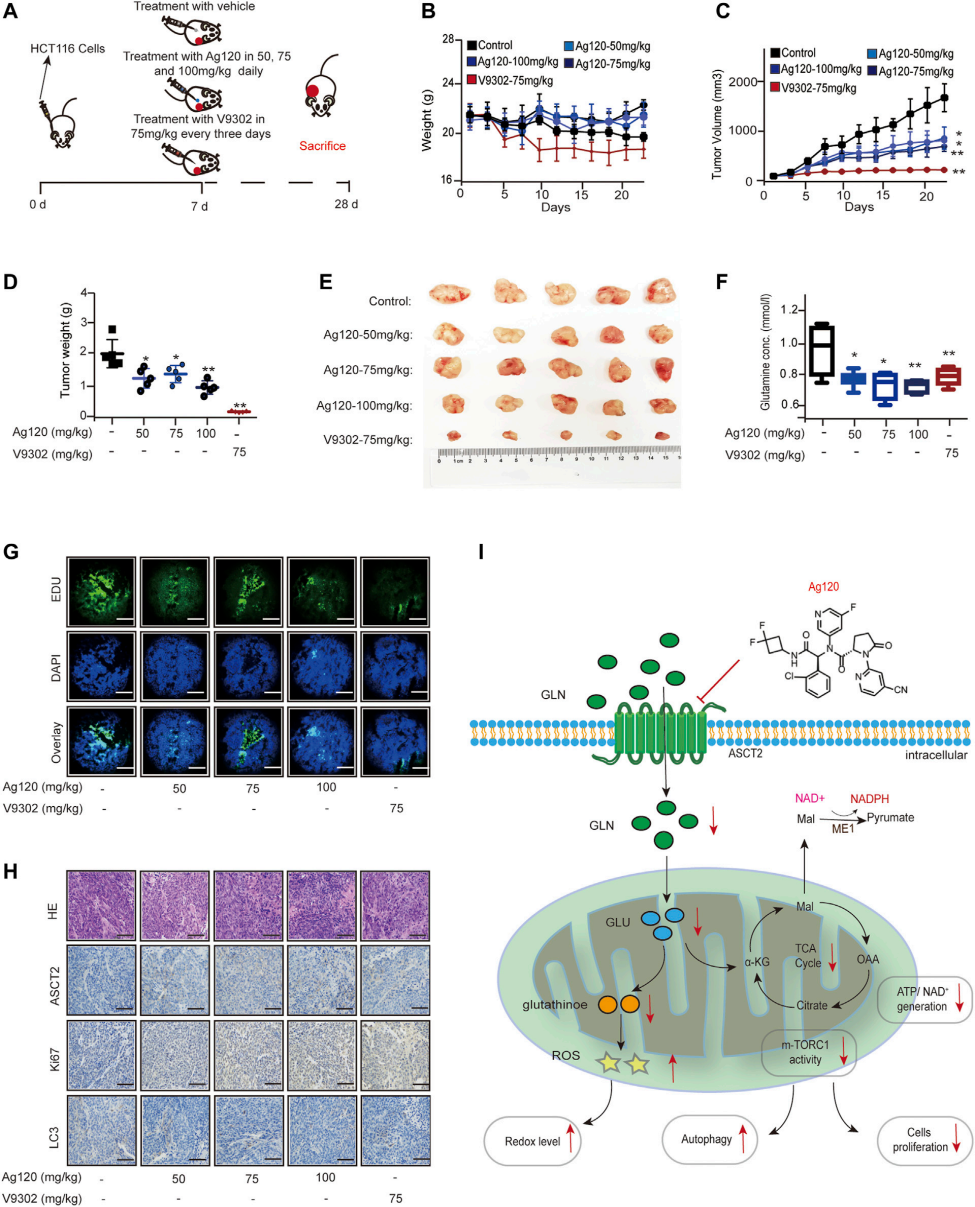
Effect of Ag120 on HCT116 xenograft growth in mice. **(A)** Experimental protocol. Groups of athymic BALB/c nude mice were injected subcutaneously with 10^6^ HCT116 cells and treatments were initiated 7 days later. Ag120 was injected once daily and V9302 was injected every 3 days *via* the intraperitoneal route. On day 28, animals were sacrificed and tumors and blood samples were collected for analysis. **(B)** Body weight change. **(C)** Tumor volumes. **(D,E)** Weights **(D)** and photographs **(E)** of tumor xenografts excised on day 28. **(F)** Blood glutamine levels on day 28. **(G)** Fluorescence microscopy images of EdU incorporation in sections of excised tumors. Scale bars, 50 µm. **(H)** Representative images of ASCT2, Ki67, and LC3 immunostaining in sections of excised tumors. Scale bars, 50 µm. **(I)** Schematic diagram of the proposed anti-tumor mechanism of action of Ag120 *via* regulation of glutamine metabolism in CRC cells. Mean ± SD of five mice per group. Symbols represent individual mice. *p < 0.05, **p < 0.01 by Student’s *t*-test.

Finally, we determined whether the effects of Ag120 on CRC cell phenotypes observed *in vitro* also translate *in vivo* using a mouse xenograft model. Groups of nude mice were injected subcutaneously with HCT116 cells. Beginning on day 7, the mice were injected with Ag120 once daily or with V9302 every 3 days until day 28, at which time the tumors were excised and analyzed (Figure 6A). Ag120 treatment was found to have no significant effect on body weight (Figure 6B) but significantly inhibited tumor volumes and weights (Figures 6C–E). V9302 also potently inhibited *in vivo* tumor growth, but it also affected the general health and body weight of the mice (Figure 6B). Analysis of blood and tumor samples on day 28 showed that Ag120 treatment significantly reduced serum glutamine levels (Figure 6F) and reduced EdU incorporation into tumor cells (Figure 6G; Supplementary Figure S6A). Finally, by quantifying the staining intensities obtained in immunohistochemical and fluorescent labeling experiments, we found that Ag120 decreased the expression of ASCT2, significantly inhibited expression of Ki67, and significantly increased expression of the autophagy protein LC3 in the tumors (Figure 6H; Supplementary Figures S6B–D). Taken together, these data demonstrate that treatment of CRC tumor-bearing mice with Ag120 effectively inhibited glutamine metabolism and augmented autophagy, thereby suppressing tumor progression *in vivo*. A model of the potential glutamine-mediated anti-tumor effects of Ag120 is shown in Figure 6I.

## Discussion

Metabolic reprograming in cancer cells provides many opportunities for therapeutic drug development and precision medicine (Hanahan and Weinberg, 2011; Koppenol et al., 2011). Cancer cells exhibit an increased demand for glutamine compared with normal cells because of their rapid metabolism and proliferation (Deberardinis and Cheng, 2010; Noel et al., 2010; Mohamed et al., 2014; Manabu et al., 2020). In this context, glutamine is a conditional essential amino acid (Liu et al., 2018). Previous studies have revealed that ASCT2 is a major glutamine transporter and is upregulated in multiple cancer cell types such as breast, gastric, prostate, and colorectal cancers (Fuchs and Bode, 2005; Miyo et al., 2016). Consequently, a large number of ASCT2-targeting small molecule drugs have been developed or discovered; however, none of them have entered clinical trials as ASCT2 inhibitors to date (Grewer and Grabsch, 2004; Esslinger et al., 2005; Canul-tec et al., 2017; Schulte et al., 2018). In the present study, we identified Ag120 as a novel ASCT2 inhibitor that had anti-tumor effects in CRC cells mediated by blockade of ASCT2-dependent glutamine transport. Most importantly, Ag120 has been approved in the United States for the treatment of several cancers and is undergoing clinical testing for other indications (Alan et al., 2014; Emadi et al., 2014; Kelly et al., 2019), which provides a wealth of information regarding the safety profile of Ag120 in healthy volunteers and cancer patients. Our study increases the spectrum of cancers that may be treatable with Ag120 and provides proof of concept for the development of ASCT2 inhibitors for cancer therapy.

Previous work has identified several ASCT2-targeting small molecule inhibitors and antibodies that block glutamine uptake and inhibit the proliferation of CRC cells (Schulte et al., 2018; Hara et al., 2020). Here, we report that the effects of Ag120 were equivalent to or exceeded those of the known ASCT2 inhibitor V9302, including inhibition of growth and survival caused by a reduction in mTOR and ERK signaling (Papa et al., 2019; Hara et al., 2020), induction of autophagy, and increased oxidative stress caused by a decrease in GSH levels (Hara et al., 2020). Importantly, the anti-tumor effect of Ag120 was also observed in a nude mouse xenograft model of CRC. The anti-ASCT2 monoclonal antibody Ab3-8 has been shown to have a weak inhibitory effect on CRC cells (Hara et al., 2020); however, this appears to be less potent than Ag120. Similarly, Ag120 fares well when compared with another glutamine uptake inhibitor, GPNA (Esslinger et al., 2005). This compound is a known ASCT2 inhibitor but exhibits poor potency and selectivity for human cells (Esslinger et al., 2005; Chiu et al., 2017). Thus, Ag120 appears to have equal or better anti-tumor effects in CRC compared with several known ASCT2 inhibitors.

ASCT2 is expressed in normal large intestinal tissue, suggesting that targeting ASCT2 as a cancer therapy might cause some side effects (Utsunomiya-Tate et al., 1996). However, recent studies showed that ASCT2/*SLC1A5*-deficient mice exhibit normal growth and survival (Nakaya et al., 2014; Masle-Farquhar et al., 2017), indicating that targeting of ASCT2 for cancer therapy is likely to be well tolerated and elicit few adverse effects. Moreover, Ag120 had little effect on the body weights of tumor-bearing mice compared with V9302 in the present study. In support of this, data from clinical trials of Ag120 in healthy volunteers and cancer patients have shown mild liver damage and other moderate adverse effects (Kelly et al., 2019). Other studies with Ag120 also indicate that the compound is well tolerated and that most adverse events are of mild to moderate severity (Alan et al., 2014; Emadi et al., 2014; Fan et al., 2015; Brandon et al., 2017).

In the present study, we observed that the Ag120-induced inhibition of glutamine metabolism was accompanied by a concomitant increase in glucose uptake, suggesting that the latter is a compensatory mechanism of energy production. Similarly, a previous study showed that V9302 induced a compensatory increase in glucose uptake in CRC cells (Hara et al., 2020). These results suggest that the anti-tumor efficacy of Ag120 could be diminished or subverted by an increase in glucose uptake and metabolism*.* Since the metabolic heterogeneity of tumor cells suggests that some are cells are dependent on both glucose and glutamine for energy production and proliferation (Vander Heiden et al., 2009; Deberardinis and Cheng, 2010; Li and Zhang, 2016). Therefore, it may be more effective to combine the therapeutic use of Ag120 with inhibitors of glucose metabolism. For example, co-treatment of breast cancer and CRC cells with V9302 and the glucose analog 2-deoxyglucose is known to increase the efficacy of V9302 (Luo et al., 2020). The results of our study suggest that combination therapy with Ag120 and glucose inhibitors might be a potential new strategy for the treatment of cancer.

Our study suggests that Ag120 is a new ASCT2 inhibitor and exerts an anti-tumor effect in CRC. However, there are no specific biomarkers for the anti-tumor efficacy of Ag120 and other ASCT2 inhibitors. It is reported that the anti-tumor activity of ASCT2 inhibitors does not necessarily correlate with the expression of transporter proteins (Schulte et al., 2018), and tumor cells that are sensitive to ASCT2 inhibitors are mainly susceptive to glutamine withdrawal (Schulte et al., 2018; Hara et al., 2020). Clinically, the anti-tumor efficacy of ASCT2 inhibitors could be quantitatively assessed by using non-invasive PET imaging of glutamine uptake (Schulte et al., 2018). However, this technology cannot measure the anti-tumor activity of ASCT2 inhibitors. Therefore, larger studies will be needed to evaluate correlations between the efficacy of Ag120 and other ASCT2 inhibitors and oncogene status and metabolic heterogeneity, and to identify biomarkers of their activity.

## Conclusion

In the past several years, interest has increased in the use of ASCT2 inhibitors as a treatment for cancer. In our study, we report for the first time that inhibition of ASCT2 function and glutamine metabolism may be a second mechanism to explain the anti-cancer effects of the IDH1mt inhibitor Ag120. We anticipate that our findings will shed light on the mechanism of Ag120 activity, extend the potential cancers that may be treatable with Ag120, and provide new insight into the potential utility of other ASCT2 inhibitors as anti-cancer agents.

## Data Availability

The original contributions presented in the study are included in the article/Supplementary Material, further inquiries can be directed to the corresponding authors.

## Supplementary Material

Supplementary Figure 1 The combination of V9302 with ASCT2 and Effect of Ag120 on glucose **uptake in CRC cells. (A)** SPR analysis of the binding affinity of V9302 for purified ASCT2. **(B)** Glucose uptake was measured after incubation with Ag120 or V9302 for 40 min.

Supplementary Figure 2 Quantifications of the indicated protein levels. The relative protein levels of ERK1 **(A)**, p-ERK1 **(B)**, p-mTOR1 b, m-TOR **(D)**, p-P70S6K **(E)** and P70S6K **(F)** in Figure 2G were quantified by Image-Pro Plus 6 software. Mean ± SD of 3 experiments. **P* < 0.05, ***P* < 0.01 by Student’s *t*-test.

Supplementary Figure 3 Quantification of autophagy induction by Ag120. **(A,B)** Autophagosome density **(A)** and lysosome density **(B)** were assessed by counting of cells labeled with MDC and LysoTracker dyes, respectively, after incubation with Ag120 or V9302 for 72 h. The relative protein levels of LC3 II **(C)**, LC3 II **(D)**, LAMP1 **(E)**, Beclin-1 **(F)**, ATG7 **(G)** and ATG5 **(H)** in Figure 3D were quantified by Image-Pro Plus 6 software. Mean ± SD of 3 experiments. **P* < 0.05, ***P* < 0.01 by Student’s *t*-test.

Supplementary Figure 4 Effects of Ag120 on CRC proliferation in glutamine-free medium. (A) The relative protein levels of IDH1 in Figure 4 **(B)** were quantified by Image-Pro Plus 6 software. **(B–F)** HCT116 and HT29 cells were treated with Ag120 (45 µM) for 72 h in DMEM medium containing 0 or 2 mM l-glutamine and then analyzed. **(B)** Cell numbers detected using a CCK-8 kit. **(C)** Colony formation quantified by light microscopy. **(D)** Relative contribution of Ag120 after glutamine depletion to inhibition of colony formation. **(E)** Quantification of EdU incorporation by fluorescence microscopy. **(F)** Relative contribution of Ag120 to inhibition of EdU incorporation after glutamine depletion. Mean ± SD of three3 experiments. **p P*< 0.05, ***p P*< 0.01 by Student’s *t*-test.

Supplementary Figure 5 Effects of Ag120 on glutamine metabolism and proliferation of CRC cells after ASCT2/SLC1A5 knockdown or overexpression. **(A,B)** RT-qPCR analysis of SLC1A5 mRNA levels in ASCT2-knockdown **(A)** and ASCT2-overexpressing **(B)** HCT116 and HT29 cells. **(C,D)** The relative protein levels of ASCT2 in Figure 5 **(A,B)** were quantified by Image-Pro Plus six6 software. **(E,F)** Glutamine uptake in ASCT2-knockdown **(E)** and ASCT2-overexpressing **(F)** HCT116 and HT29 cells treated with Ag120 (15 µM) for 2 h. **(G,H)** As described for **(E,F)** except cell numbers were quantified using a CCK-8 kit. **(I,K)** Colony formation by ASCT2-knockdown **(I)** and ASCT2-overexpressing **(K)** HCT116 and HT29 cells after Ag120 (45 µM) treatment for 10 days. **(J,L)** Relative contribution of Ag120 treatment to inhibition of colony formation in ASCT2-knockdown **(J)** and ASCT2-overexpressing **(L)** cells. Mean ± SD of 3 experiments. **p P*< 0.05, ***p P*< 0.01 by Student’s *t*-test.

Supplementary Figure 6 Quantification of Ag120-mediated inhibition of ASCT2 expression, proliferation, and autophagy in HCT116 xenografts *in vivo*. **(A–D)** Figure 6A for experimental protocol. Tumors were excised on day 28 and positively stained cells were quantified. **(A)** EdU-incorporating cells measured by fluorescence microscopy. **(B–D)** Intensity of immunohistochemical staining of ASCT2 **(B)**, Ki67 **(C)**, and LC3 **(D)** expression. Mean ± SD of 3 experiments. **P <* 0.05, ***P* < 0.01 by Student’s *t*-test.

## Abbreviations

ASCT2, alanine, serine, cysteine-preferring transporter 2; SLC1A5, solute carrier family 1, member 5; CRC, colorectal cancer; ATP, adenosine triphosphate; ROS, reactive oxygen species; NADH, nicotinamide adenine dinucleotide (reduced); Ag120, ivosidenib; TCA, tricarboxylic acid; α-KG, 2-ketoglutaric acid; Glu, glucose; Gln, glutamine
