# Supplementary figures and images for: Ag120-Mediated Inhibition of ASCT2-Dependent Glutamine Transport has an Anti-Tumor Effect on Colorectal Cancer Cells
Source: Front Pharmacol. 2022 Mar 28;13:871392. doi: 10.3389/fphar.2022.871392 (PMC8996072; doi:10.3389/fphar.2022.871392)

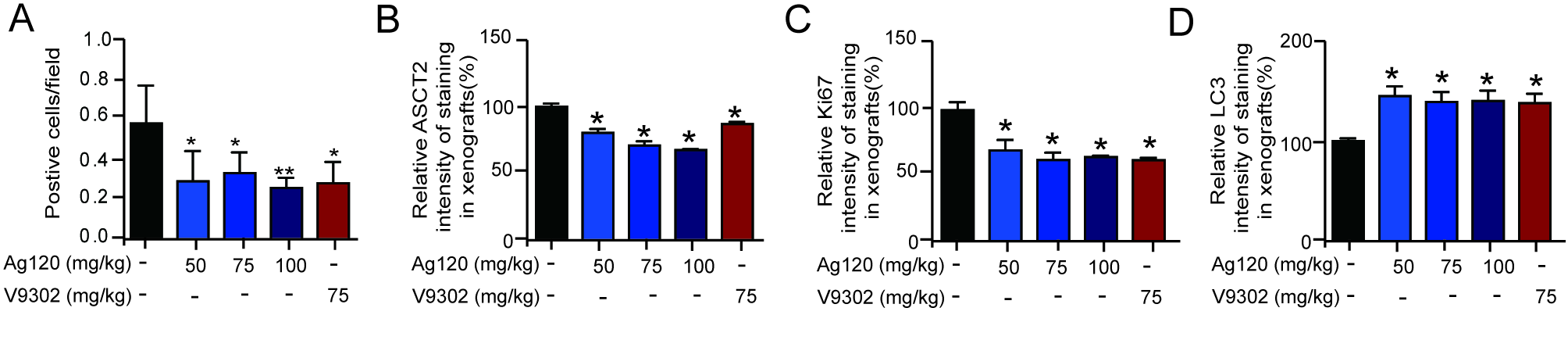

Supplement: Supplementary file 1 [file Image6.tif]

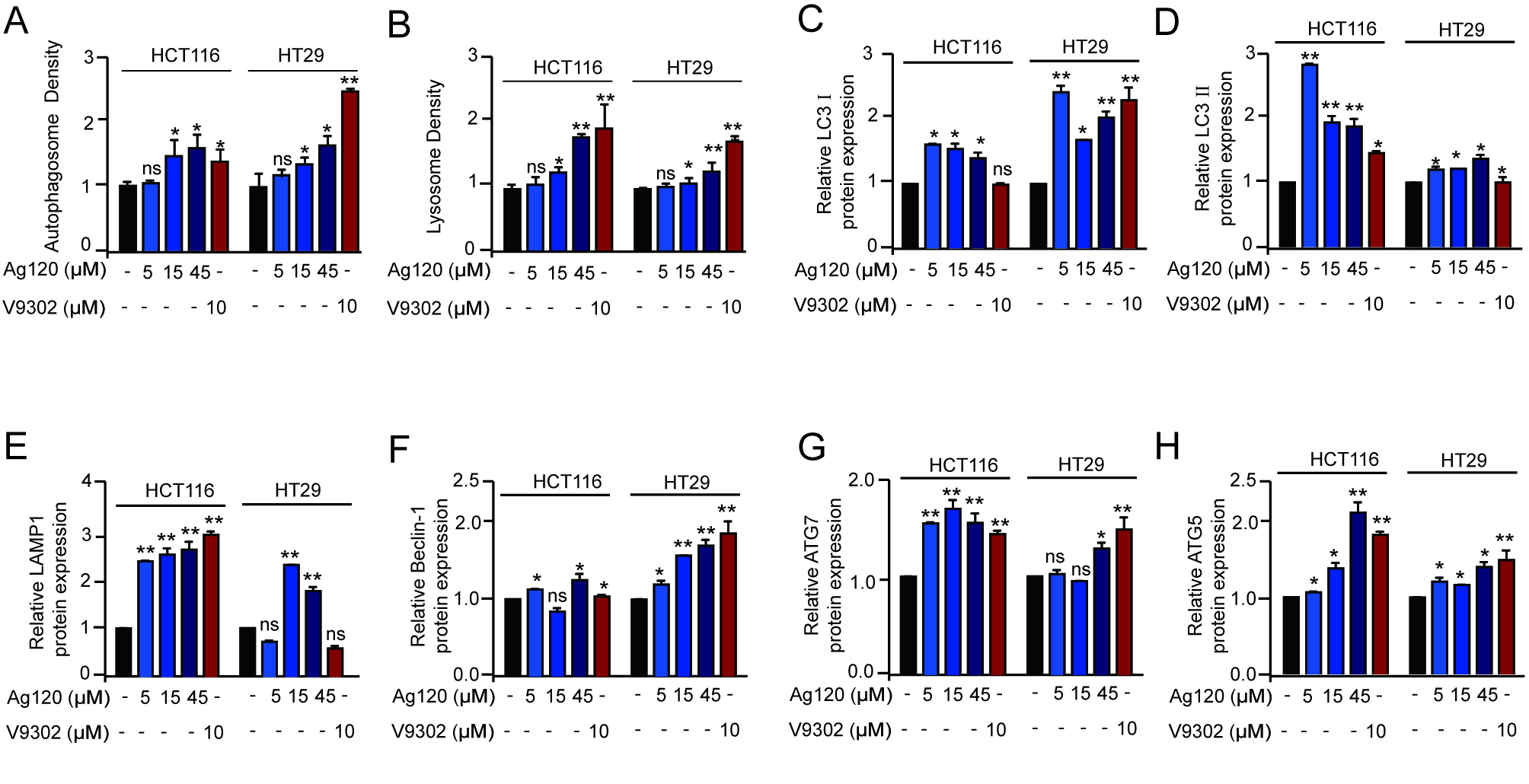

Supplement: Supplementary file 2 [file Image3.tif]

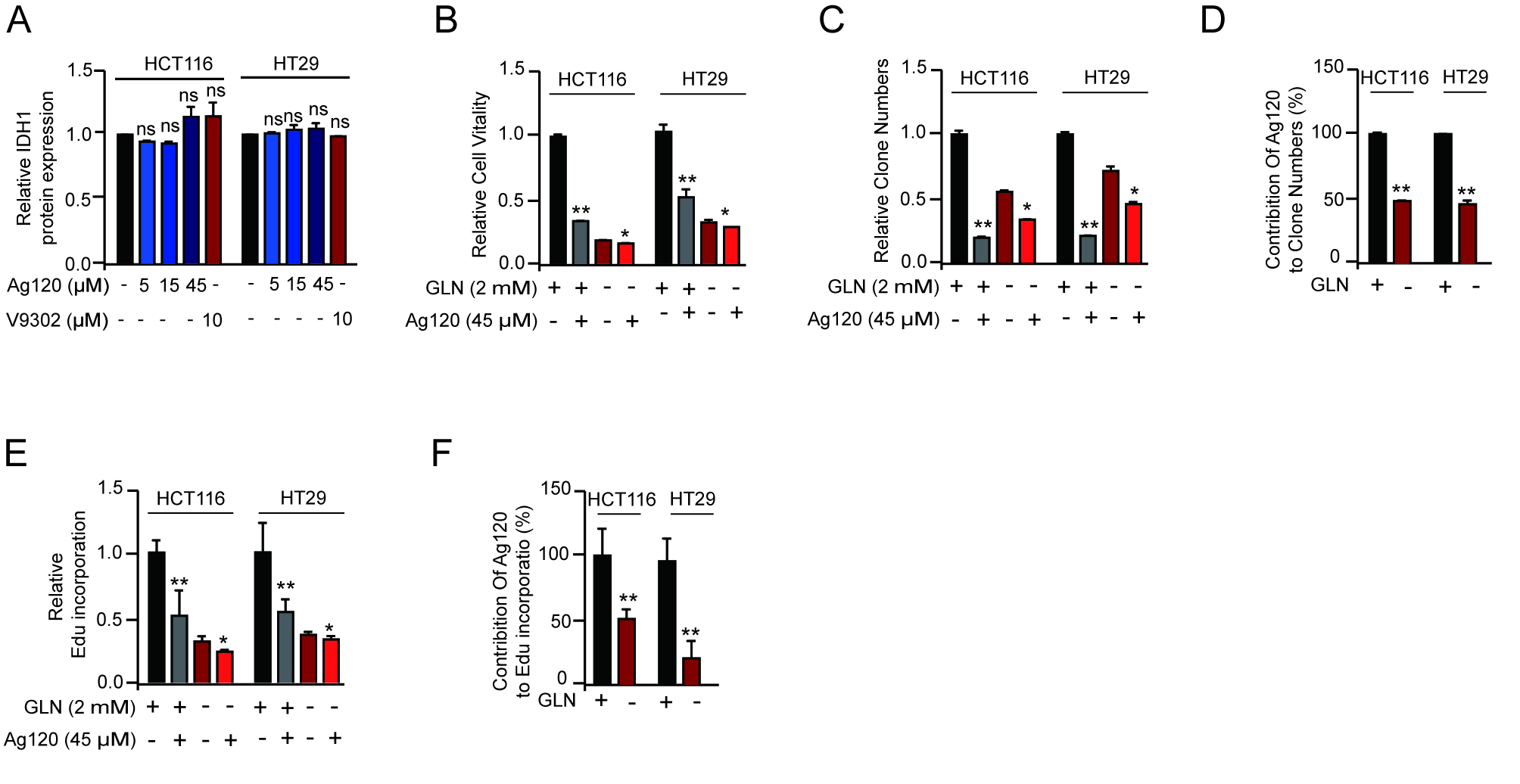

Supplement: Supplementary file 3 [file Image4.tif]

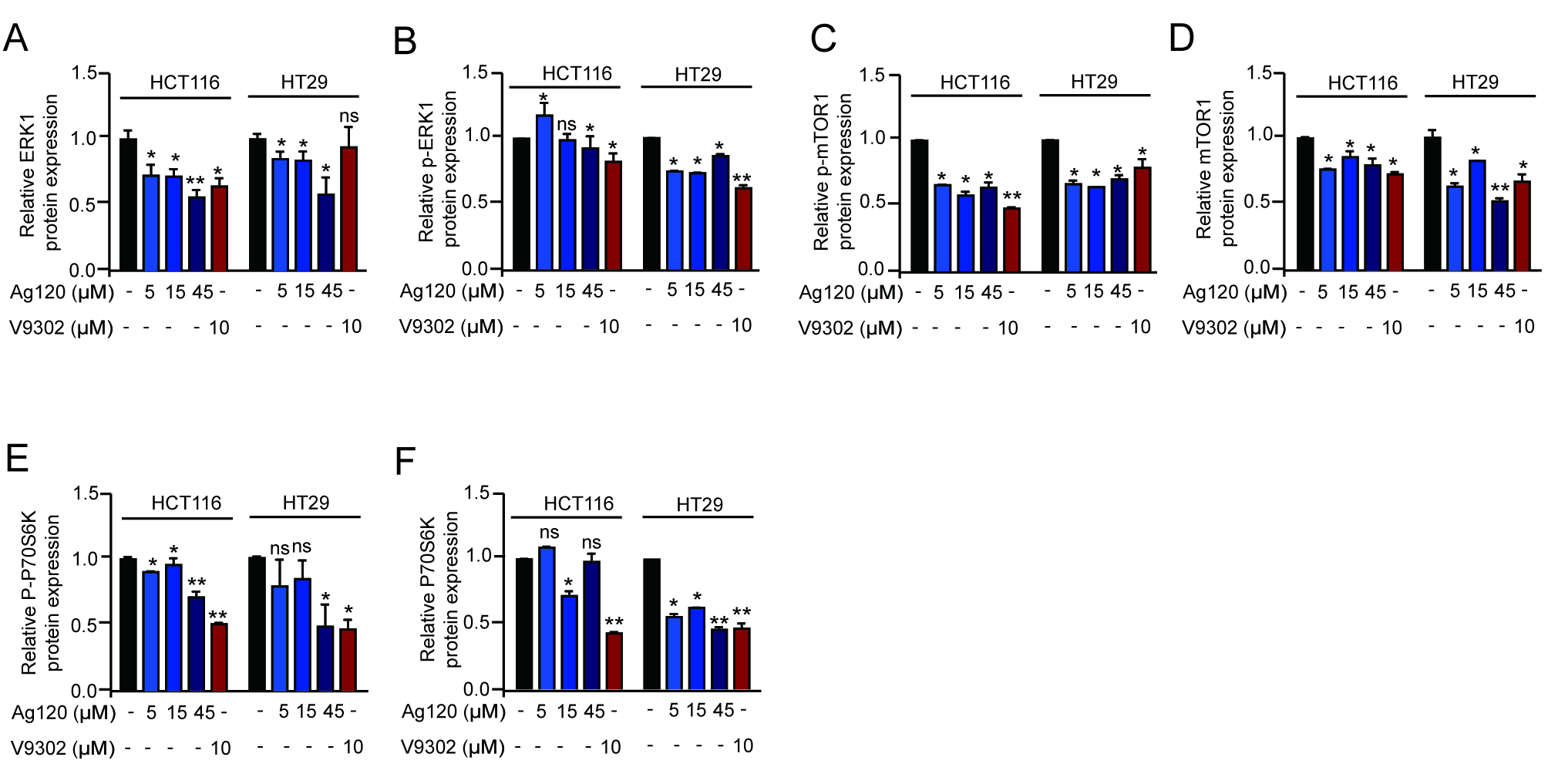

Supplement: Supplementary file 4 [file Image2.tif]

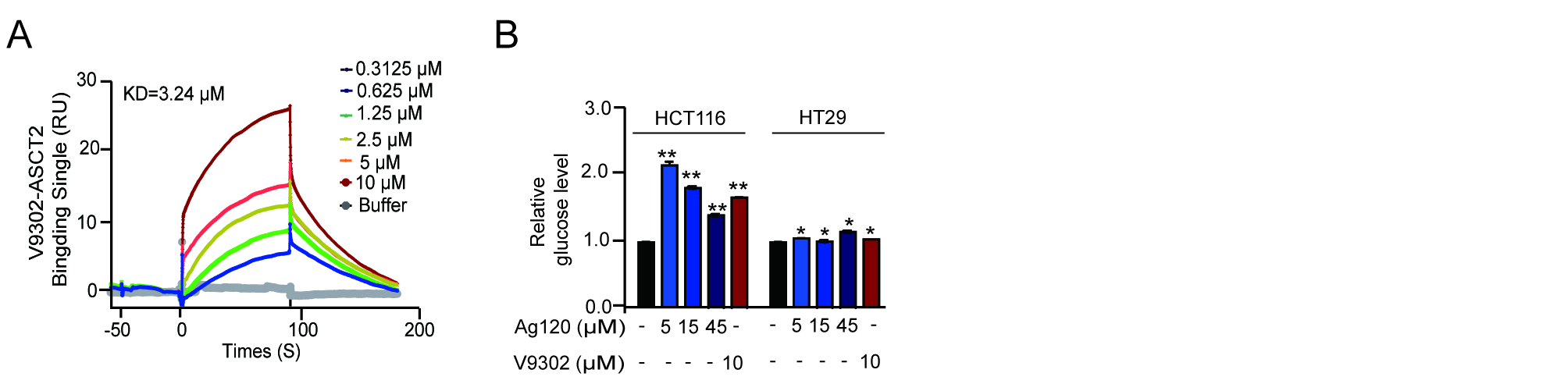

Supplement: Supplementary file 5 [file Image1.tif]

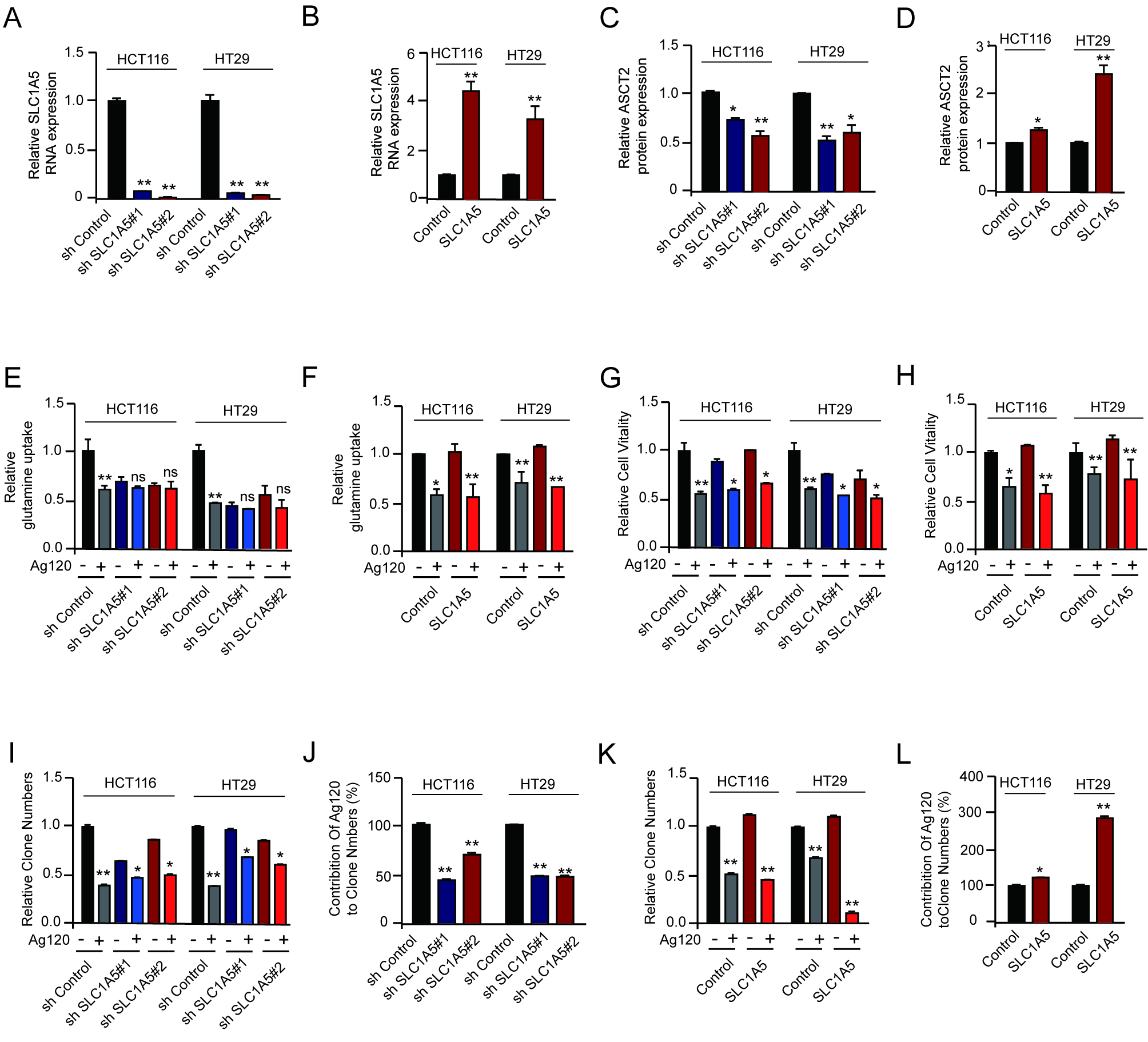

Supplement: Supplementary file 6 [file Image5.tif]
